# Experience with 10 years of a robotic surgery program at an Academic Medical Center

**DOI:** 10.1007/s00464-021-08478-y

**Published:** 2021-04-12

**Authors:** Sarah B. Stringfield, Lisa A. Parry, Samuel G. Eisenstein, Santiago N. Horgan, Christopher J. Kane, Sonia L. Ramamoorthy

**Affiliations:** 1 Department of Surgery, University of California San Diego, 9300 Campus Point Drive, La Jolla, CA 92037-7400 USA; 2 Department of Urology, University of California San Diego, 200 W. Arbor Drive #8897, San Diego, CA 92103-8897 USA; 3 6907 Stefani Drive, Dallas, TX 75225 USA

**Keywords:** Robotic Surgery, Operative time, Readmission, Costs

## Abstract

**Background:**

Few studies have examined robotic surgery from a programmatic standpoint, yet this is how hospitals evaluate return on investment clinically and fiscally. This study examines the 10-year experience of a robotic program at a single academic institution.

**Study design:**

All robotic operations performed at our institution from August 2005 to December 2016 were reviewed. Data were collected from the robotic system and hospital databases.

**Results:**

A total of 3485 robotic operations were performed. Yearly case volume nearly quadrupled. There have been 37 robotic-trained surgeons in 5 specialties performing 53 different operations. Rate of conversion to open was 4.2%. American Society of Anesthesiologists (ASA) class increased over time, with ASA class 3 increasing from 20% of patients to 45% of patients.

Average case time in 2005 was 453 min, but decreased by 46% to 246 min by 2007, then remained relatively stable (range 226–247). Operating efficiency improved, with room time and case time decreasing by 9% in the past 4 years.

Average cost for robotic supplies was $1519 per case. Additional costs per case related to equipment and contracts totaled an average of $11,822.

Average length of stay (LOS) for robotic cases was 3.3 days, compared to 3.0 days for laparoscopic and 7.0 for open. Cost per day for admission after robotic surgery was 1.7 times greater than the cost of open or laparoscopic surgery. Total admission costs of robotic operations were 1.5 times those of laparoscopic surgery, but less than open operations. Readmissions following robotic cases were lower than open (15% v 26%, *p* < 0.0001).

**Conclusions:**

Over 10 years, the use of robotic technology has grown significantly at our institution, with good fiscal and clinical outcomes. Operating room costs are high; however, efficiency has improved, LOS is shorter, admission costs are lower than open operations, and readmission rates are lower.

In its short history, robotic-assisted surgery has been a rapidly growing and changing field. In 1994, Computer Motion, Inc. (Goleta, CA) developed the first robot to assist in laparoscopic surgery, the Automated Endoscope System for Optimal Positioning (AESOP) robot, which could maneuver an endoscope inside the human body and was controlled by voice commands or a computer [1]. This was quickly replaced by the ZEUS system that was developed in 1995 and approved by the Food and Drug Administration (FDA) in 2001 [2]. This robotic system had multiple arms and was able to hold 28 different instruments. Intuitive Surgical (Sunnyvale, California) developed a prototype of their da Vinci system in 1997, and it was FDA approved for general laparoscopic surgery in 2000 [3]. Since its introduction, it has replaced most other robotic surgical systems and is the most widely used robotic surgical system in use throughout the world [4].

Utilization of the da Vinci system has increased significantly since its introduction. There were 80,000 robotic procedures performed in 2007, and in 3 years the number of yearly procedures nearly tripled, to 205,000 in 2010 [5]. In 2013, more than 500,000 robotic procedures were performed, and a total of more than 3 million robotic procedures have been performed [5]. Additionally, there are a large number of da Vinci systems throughout the United States and the world. As of September 2016, there were 3800 units worldwide and 2500 units in the United States [5].

There have been thousands of articles published on robotic surgery. Literature review will demonstrate that the vast majority of these studies focus on a specific procedure or surgical specialty, with the purpose of establishing safety, feasibility, and economics of the robotic procedure compared to an open or laparoscopic approach. There have been few studies that have looked at robotic surgery from a programmatic standpoint. However, this is how many hospitals evaluate return on investment both clinically and fiscally. Reviewing a robotic surgery program as a whole has advantages over reviewing specific operations individually. While looking at individual operations, some robotic operations are more expensive, have longer length of stay, or higher readmission rates compared to equivalent open or laparoscopic operations, while some robotic operations show an advantage over traditional platforms. Looking at the entire program, rather than each type of operation individually, allows us to see if the purchase of a robotic system is a good overall investment for a health system.

Robotic-assisted surgery is a technology with unique issues concerning costs and scheduling. Outcomes data in robotic surgery have been published as quickly as new technology has been introduced, causing rapid evolution of the technology and techniques based on short-term outcomes. Surgeons and institutions need to quickly adapt as this information and technology becomes available. Due to the novelty of this technology, little is known about the phenotype of robotic surgery programs as a whole and how they have evolved throughout their short lifespan.

Our institution, the University of California San Diego (UCSD) Health System, acquired our first da Vinci robotic surgical system in 2005. As we reached 10 years of having an established robotic program, we performed a review of the evolution of robotic surgery at our institution. This study will look at over 10 years of robotic surgery data from a single academic institution, including operative volume, perioperative data, costs, and admissions outcomes. This study will give a picture of the robotic program as a whole, and show how the program has grown and changed over time.

## Methods

### Data collection

All robotic-assisted operations performed at the University of California San Diego Health System between August 2005 and December 2016 were reviewed. Cases which were scheduled as robotic-assisted but in which the robot was not docked were excluded from this study. This includes those performed laparoscopically without robotic assistance or converted to open prior to docking.

Data were collected from our institution’s electronic surgical scheduling and resource management systems. This included Operating Room Scheduling Office System (ORSOS, Per-Se Technologies, Atlanta, GA) from August 2005 to October 2013 and Epic OpTime (Epic Systems Corporation, Verona, WI) from October 2013 to December 2016. Data collected by Intuitive Surgical via the da Vinci robotic surgery console or internal databases included operative minutes using the console and costs for robotic surgery supplies per case. The Institutional Review Board (IRB) determined that IRB approval was not required for this study as no identifying patient information was used.

#### Costs

Operating Room supply cost data were only available for cases scheduled with OpTime. Cardiac cases were excluded from supply cost data analysis due to significantly higher supply costs associated with implants and perfusion equipment. Costs for the robotic systems, service contracts, and nondisposable supplies were calculated from purchasing data.

#### Admissions and readmissions

Admissions data from fiscal years (July–June) 2009–2015 were reviewed for robotic operations and their equivalent open or laparoscopic operations. Robotic cases were matched to their equivalent open or laparoscopic operation and sorted by ICD9 code. All cases performed during the study time period were included. This included the following procedures and designated codes: adrenalectomy (7.22), mitral valvuloplasty (35.12), atrial septal defect repair (35.61), coronary artery bypass graft (36.15), regional lymphadenectomy (40.3), esophagectomy (42.4, 42.41), esophagomyotomy (42.7), gastrectomy (43.7, 43.99), fundoplication (44.67), small bowel resection (45.62), total colectomy (45.83), permanent ileostomy (46.23), ileostomy revision (46.41), abdominoperineal resection (48.51), rectal resection (48.62, 48.63, 48.69), proctopexy (48.76), cholecystectomy (51.23), hiatal hernia repair (53.71), lysis of adhesions (54.51, 54.59), partial nephrectomy or decapsulation (55.4, 55.91), nephroureterectomy (55.51), ureteral procedure (55.87, 56, 56.41, 56.74), cystectomy (57.49, 57.6, 57.71), prostatectomy (60.4, 60.5, 60.69), oophorectomy ± salpingectomy (65.01, 65.39, 65.41, 65.49, 65.61), hysterectomy (68.41, 68.49, 68.51, 68.59, 68.71, 69.19), and sacrocolpopexy (70.78). Information regarding robotic operations was available for ICD9 codes listed above, and either an open or laparoscopic equivalent. A small number of operations had information regarding all 3 platforms: open, laparoscopic, and robotic.

Readmissions are all-cause readmissions for 90 days following surgery. Readmissions were reported based on the total number of readmissions rather than the number of patients that had a readmission. Therefore, if a single patient was readmitted 2 times after a procedure, there were 2 readmissions for a readmission rate of 200%.

### Statistical analysis

Continuous variables were analyzed using two-tailed t-test for two independent means or analysis of variance (ANOVA) test. Categorical variables were analyzed using Chi-square test for independent variables. *P*-value less than 0.05 was considered significant.

## Results

### Robotic systems

The UCSD Health System consists of two main hospital campuses. At the Hillcrest campus, an S model was gifted to the University in 2006 and used until 2015, when it was replaced by an Si model. At the La Jolla campus, an S model was leased in 2005 and subsequently purchased in 2006. This was replaced by an Si model in 2012. A second Si system was purchased in 2010 and replaced with a dual console system Xi model in 2015. An Si video upgrade was purchased in 2012 and an Si skills simulator was purchased in 2012.

### Case volume

**Fig. 1:**
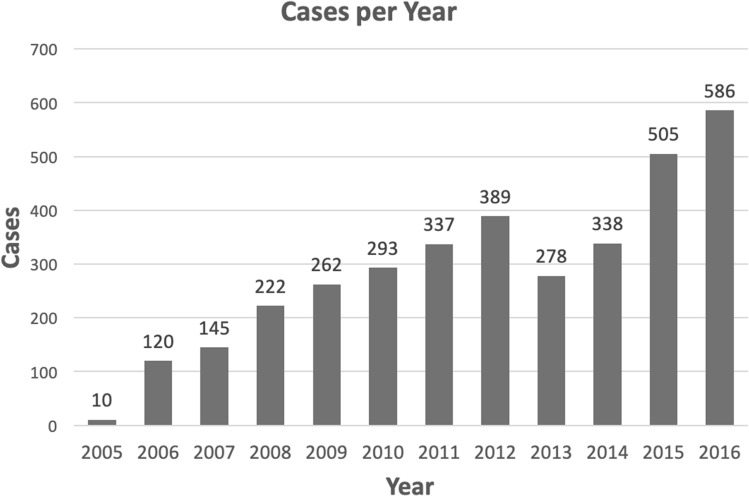
Total cases per year

A total of 3485 robotic-assisted operations were performed. This ranged from 120 in 2006 to 586 in 2016 (Fig. 1).

**Fig. 2:**
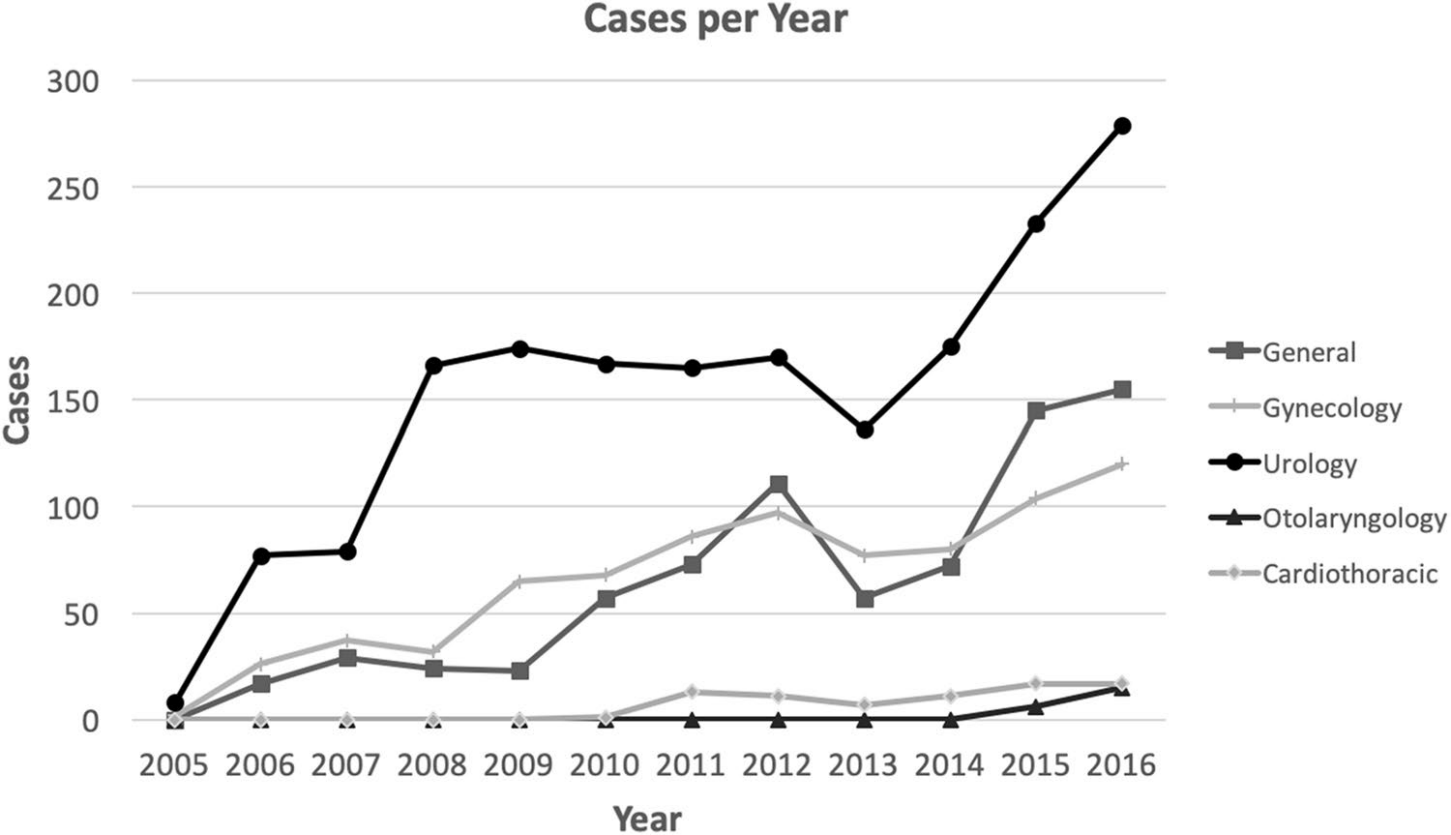
Cases per year by specialty

The majority of cases were performed by Urology (1829, 52%), followed by Gynecology (794, 23%), General Surgery (763, 22%), and small numbers of cases performed by Cardiothoracic Surgery (77, 2%) and Otolaryngology (21, 1%) (Table 1). Volume per year by specialty is shown in Fig. 2. There were a large number of unique attending surgeons performing robotic surgery, including 37 different surgeons overall and 28 in 2016. Each specialty increased the number of attending surgeons performing robotic surgery over the past 10 years (Table 1).

**Table 1:** Specialty case volume and attending surgeon volume by year

Year	Cardiothoracic		General		Gynecology		Otolaryngology		Urology	
	Cases	Surgeons	Cases	Surgeons	Cases	Surgeons	Cases	Surgeons	Cases	Surgeons
2005	0	0	0	0	2	2	0	0	8	2
2006	0	0	17	5	26	4	0	0	77	3
2007	0	0	29	6	37	5	0	0	79	5
2008	0	0	24	5	32	4	0	0	166	3
2009	0	0	23	3	65	6	0	0	174	6
2010	1	1	57	3	68	6	0	0	167	7
2011	13	2	73	4	86	7	0	0	165	6
2012	11	2	111	4	97	7	0	0	170	4
2013	7	1	57	5	77	9	0	0	136	5
2014	11	1	72	4	80	5	0	0	175	7
2015	17	2	145	6	104	6	6	3	233	7
2016	17	3	155	7	120	6	15	3	279	9
Total cases or number of unique surgeons	77	3	763	10	794	10	21	3	1829	11

There was large diversity in the types of cases performed. A total 53 different surgical procedures were performed across the 5 specialties. While there was diversity in types of cases performed, the majority of operations were within the pelvis. The top 3 most commonly performed operations included prostatectomy, hysterectomy, and proctectomy, and accounted for 56% (1969/3485) of total case volume.

### Operative time

**Fig. 3:**
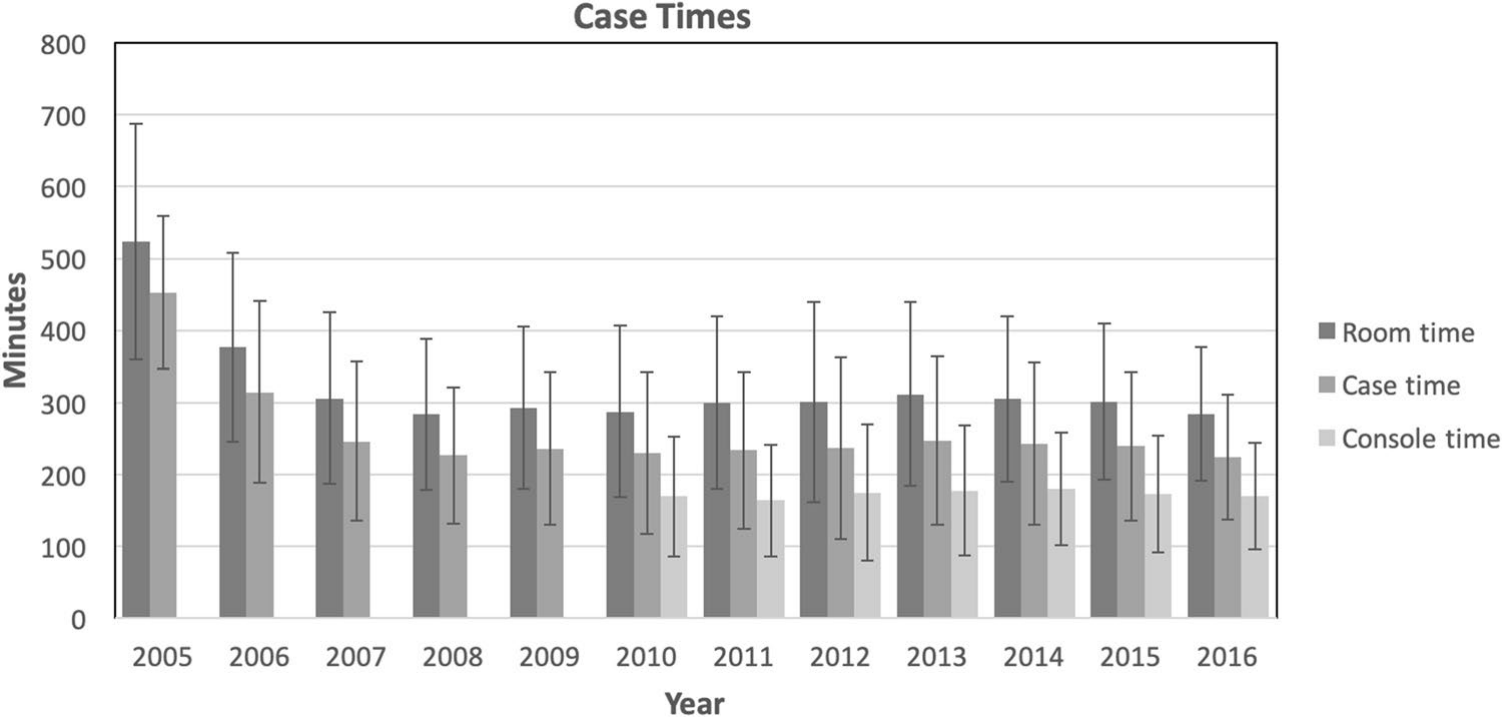
Case times per year. Error bars indicate standard deviation

Case time decreased in the first 3 years of the robotic surgery program as operating room (OR) personnel and surgeons became more familiar with the system. Case time decreased 31% in the first year from 453 to 314 min, and an additional 22% between 2006 and 2007 (314 to 246 min). Case time remained fairly steady from 2007 to 2016, with an average of 236 min. However, there was a small decrease yearly from 2013 to 2016, for an overall additional decrease of 23 min during that time period (9% of operative time in 2013) [Table 2; Fig. 3]. The shortest average operative time was 224 min in 2016. Operative time is consistently 78–80% of room time after the first 2 years. Room time also decreased over the past 4 years, with a total decrease of 27 min (8.8%). Robotic console time data were available starting in 2010. Console time accounted for 55–60% of total operative room time [Table 2] and was on average 173 min (± 83.6) per case.

**Table 2:** Case times

Year	Room time		Case time			Console time		
	Minutes	Standard deviation	Minutes	Standard Deviation	% of OR time	Minutes	Standard Deviation	% of OR time
2005	524.0	164.2	453.0	106.2	86			
2006	377.0	130.9	314.2	126.3	83			
2007	305.6	119.2	246.1	110.2	81			
2008	283.6	104.3	226.5	94.4	80			
2009	292.4	114.0	236.0	106.2	81			
2010	287.3	119.2	229.8	112.2	80	169.5	83.8	59
2011	299.3	119.6	233.7	109.2	78	163.8	77.9	55
2012	300.8	139.3	236.6	126.5	79	174.8	94.4	58
2013	311.8	128.0	247.3	116.6	79	177.6	90.7	57
2014	304.9	114.6	242.8	113.0	80	180.0	78.32	59
2015	301.0	108.6	239.2	103.7	79	172.5	81.0	57
2016	284.4	92.8	224.3	86.5	79	169.9	73.86	60

Room time calculated from time entering room to time leaving room

Case time was calculated from incision to closure

**Fig. 4:**
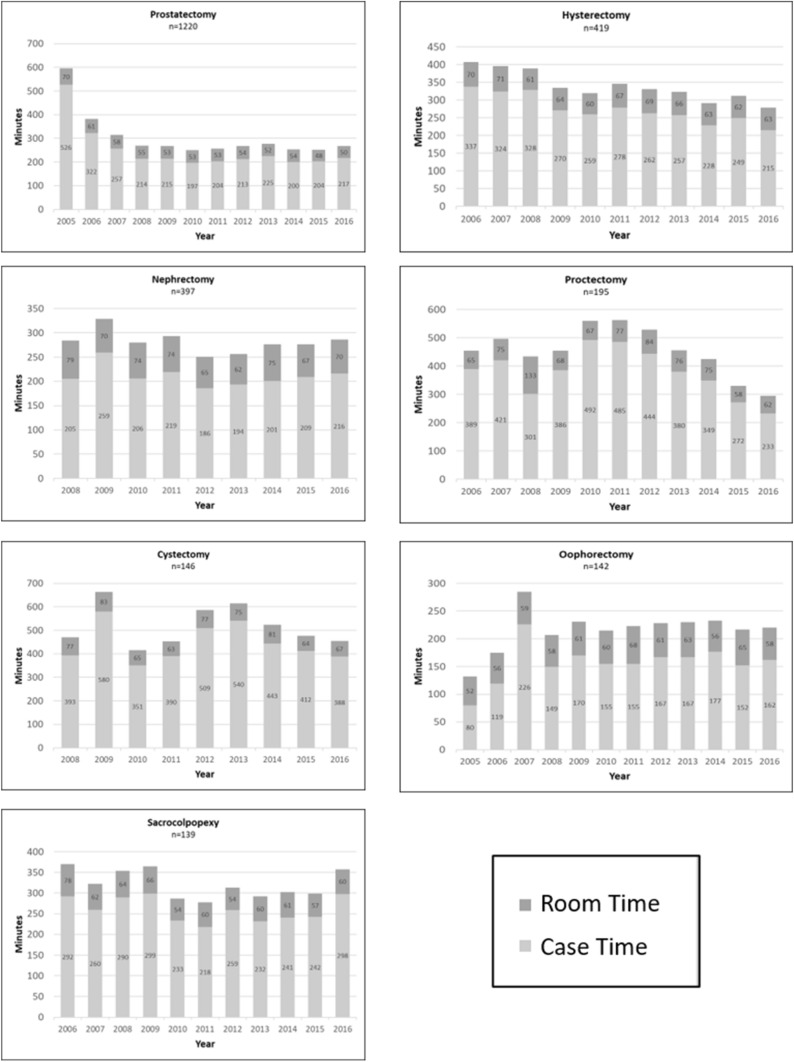
Case times for most common operations. Light gray bar represents operative time. Dark gray bar is additional minutes spent in operating room. Total height of bar is Room Time

Operative Time and Room Time for specific operations is shown in Fig. 4. Operations in which > 100 cases were performed during the study time period were included and we excluded cases that were converted to open. The operations included were prostatectomy, hysterectomy, nephrectomy (partial, radical, and donor), proctectomy (proctocolectomy, low anterior resection, and abdominoperineal resection), cystectomy, oophorectomy (oophorectomy, salpingo-oophorectomy, and ovarian cystectomy), and sacrocolpopexy. These operations totaled 2658 cases, which are 76% of total cases. Despite high volume, the cases do not all show a linear decline in case time over the years. Prostatectomy has been relatively stable after an initial decline in case time. Hysterectomy, proctectomy, and cystectomy did not show linear declines initially, but have shown improvement in case times during the last few years of this study. There was not a clear trend of improvement in case time over the study time period with nephrectomy, oophorectomy, and sacrocolpopexy.

Additional nonoperative time in the OR (Room Time) is shown as the difference in OR Time minus Operative Time. This was relatively consistent across the different operations, with an average of 66 min per case. Average for prostatectomy was 55 min (range 48–70), hysterectomy = 65 (range 60–71), nephrectomy = 71 (range 62–79), proctectomy = 76 (range 58–133), cystectomy = 72 (range 63–83), oophorectomy = 60 (range 52–68), and sacrocolpopexy = 61 (range 54–78). There was a mild decrease by an average of 8 min in room time when comparing the difference in average room time during the first 3 years performing the operation to the last 3 years within the study time period.

### Conversion to open

**Fig. 5:**
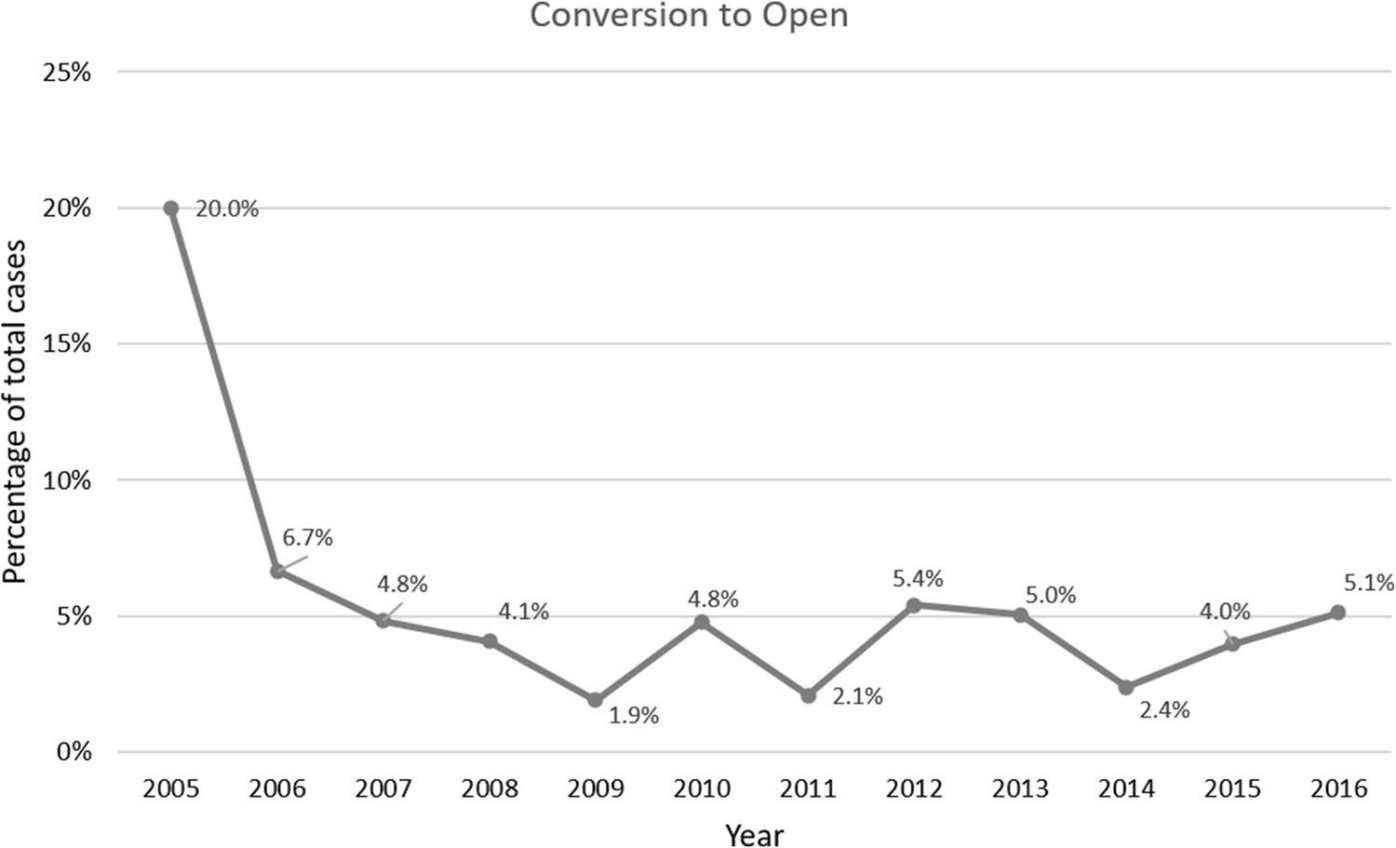
Yearly rate of conversion to open operation

Overall, 4.2% of robotic-assisted operations were converted to open. The highest percentages were in 2005 (20%, 2/10) and 2006 (6.7%, 8/120). From 2007 to 2016 the conversion rate ranged from 1.9 to 5.4% (*p* = 0.01) [Fig. 5]. Overall conversion rates varied by specialty: Urology 2.3%, General Surgery 4.5%, Cardiothoracic 5.2%, and Gynecology 8.1% (*p* < 0.001).

### Patient ASA class

**Fig. 6:**
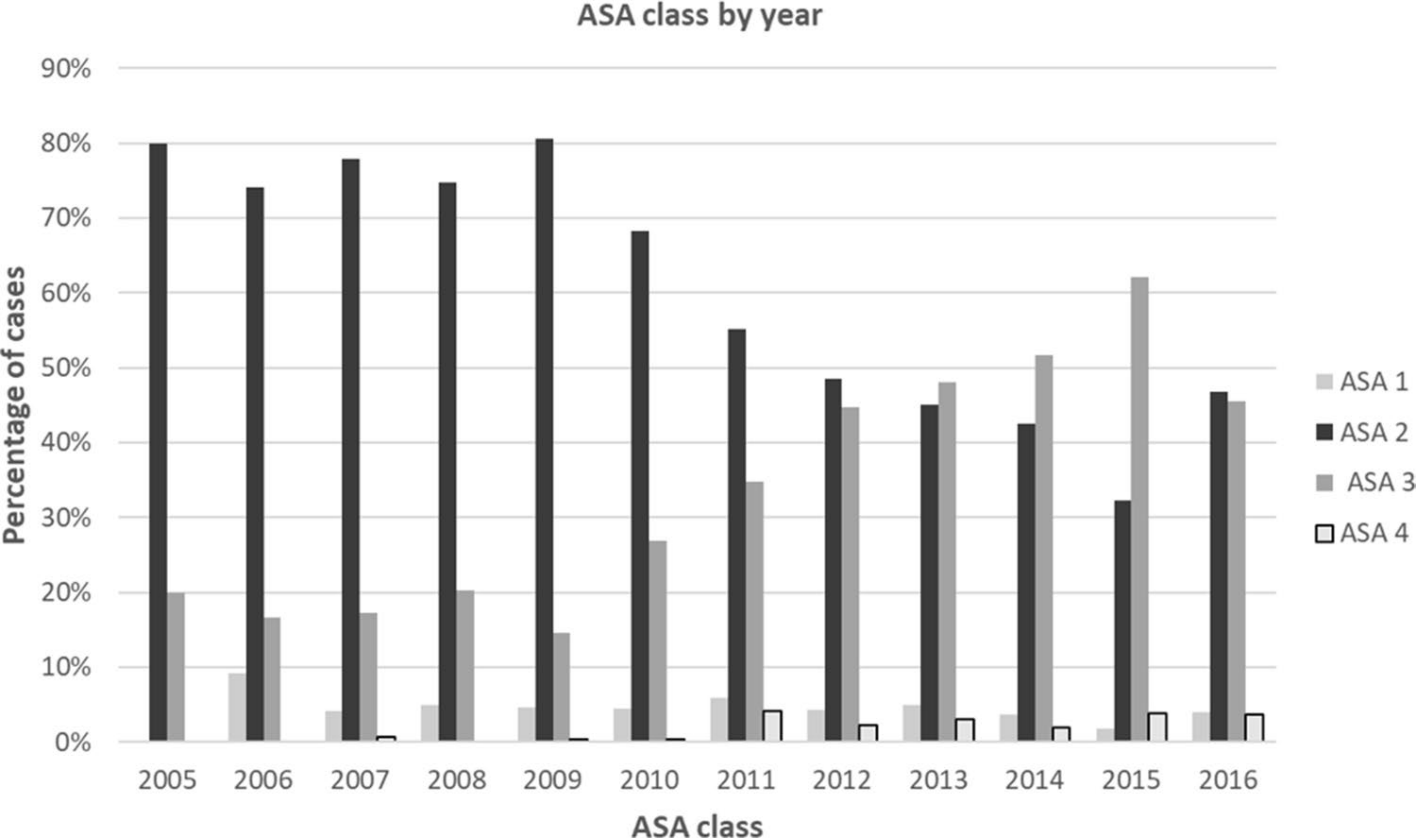
ASA class of patients. *ASA* American Society of Anesthesiologists

The American Society of Anesthesiologists (ASA) Physical Status Classification System was documented for each patient. The ASA class of patients is shown in Fig. 6 as a percentage of the cases performed each year. The distribution of patients within the ASA classes each year was statistically significant (*p* < 0.0001). The proportion of patients with higher ASA class increased over time. Class 3 patients increased from ≤ 20% of patients in 2005–2009 to > 45% of patients in 2012–2016, with an associated decrease in Class 2 patients from ≥ 74% to < 50% during the same time frames.

### Length of stay (LOS)

**Fig. 7:**
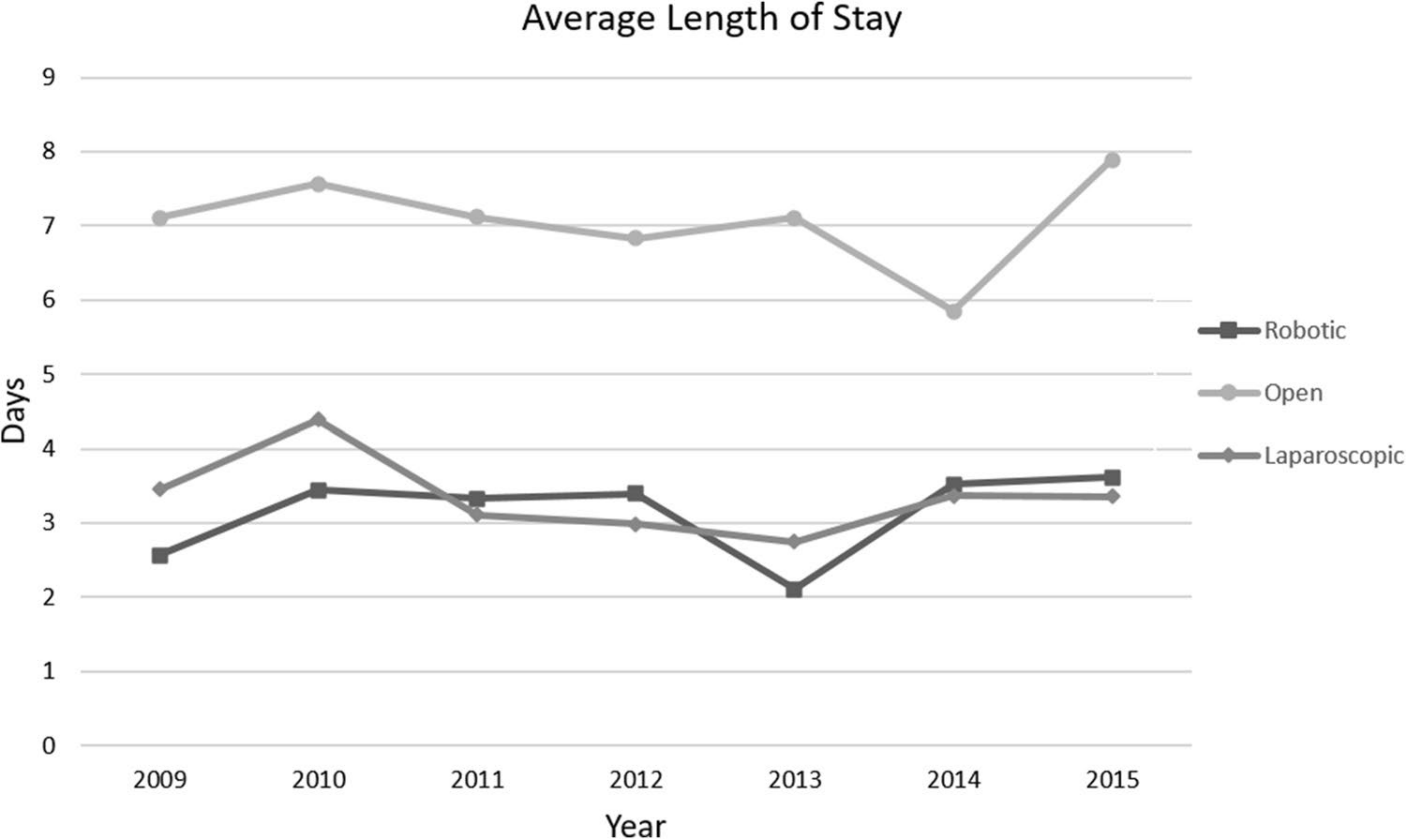
Yearly average length of stay

Admissions data were collected from 1525 robotic operations and 4363 nonrobotic operations from 2009 to 2015. When total days in hospital divided by robotic case volume was calculated, overall average LOS after a robotic surgery was 3.0 days. Median average length of stay was 2.9 days (range 1.06–11.28). Laparoscopic procedures had an overall average LOS of 3.3 days and median average LOS of 1.9 days (range 1.4–8.4). Open cases had an overall average LOS of 7.0 days and median average LOS of 5.8 days (range 1.5–29.0). Yearly average LOS for robotic cases ranged from 2.1 days to 3.6 days, with the two highest values late in this study, in fiscal years 2014 and 2015 [Fig. 7]. Yearly average LOS for laparoscopic cases ranged from 2.7 to 4.3 days, with the two highest values early in this study, in 2009 and 2010. This may reflect the transition of difficult laparoscopic cases to the robotic platform. Yearly average LOS for open cases ranged from 5.8 to 7.8 days.

### Readmissions

**Fig. 8:**
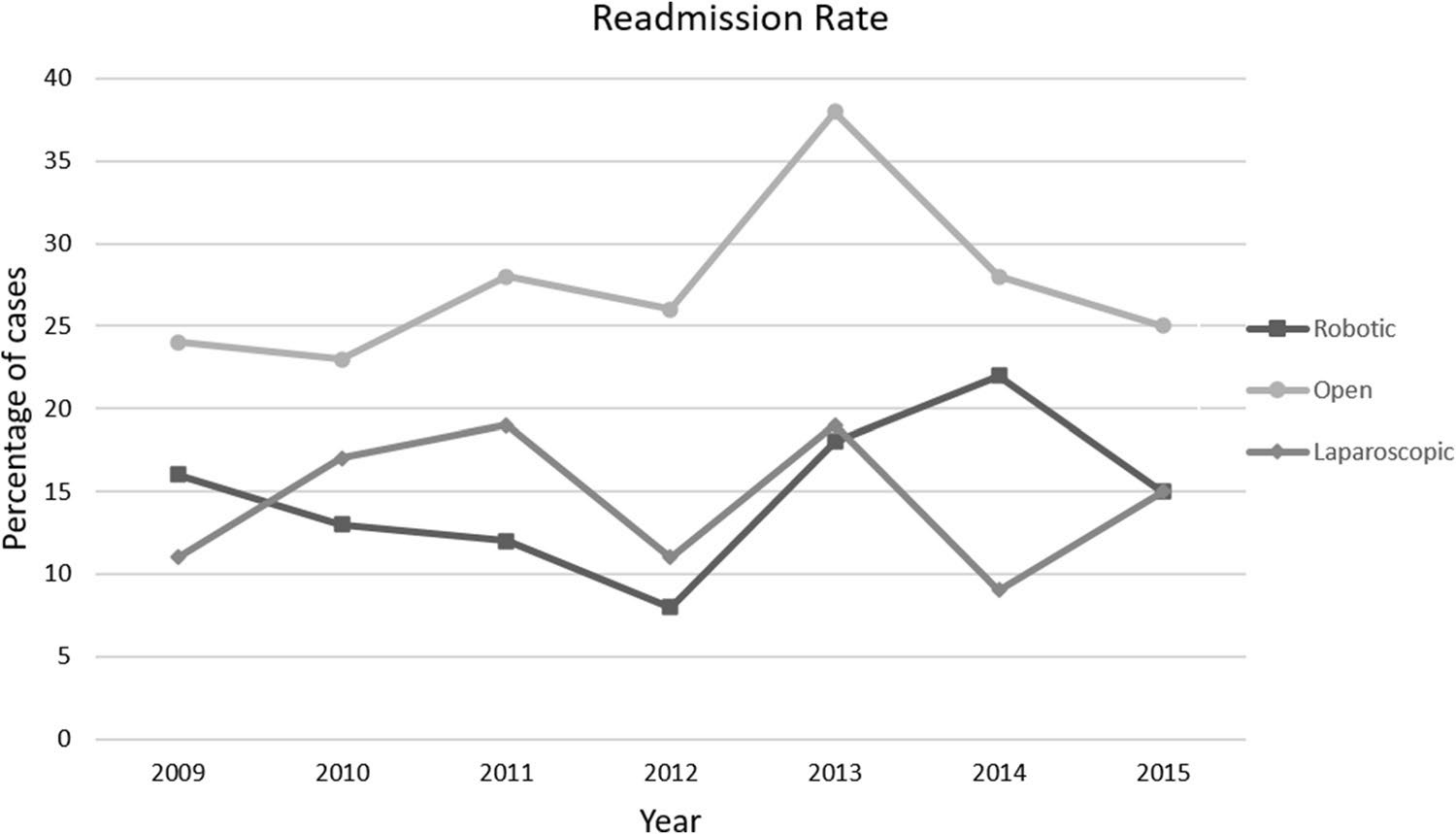
Yearly readmission rate

Readmission rate for robotic cases was 15% (214/1411). Readmission rate for laparoscopic cases was 15% (143/959) and open cases was 26% (671/2610). Yearly rate according to surgical platform is shown in Fig. 8. Robotic readmission rate was lower than laparoscopic early in the study period but became higher than laparoscopic in 2014. Readmission rate for open cases was statistically higher than for laparoscopic or robotic operations (*p* < 0.0001).

### Costs

#### Operative costs

Cost for operating room supplies, excluding cardiac cases, was $1806–$5773 per case. The median cost for robotic supplies per case was $1519 (range $809–$2178). Robotic supplies accounted for a median of 44% of overall operating room supply costs per case, but varied significantly, from 18% (esophagectomy) to 70% (transanal resection of rectal lesion) of supply costs.

Total yearly spending on nondisposable robotic instruments, parts, and repairs was divided by yearly case volume. An additional median cost of $2242.60 (range $1795.73–$2874.65) was calculated for each case for nondisposable supplies and parts.

Yearly total costs for service contracts divided by case volume accounted for a range of $6443.69–$20,135.23 per case for 2007–2016. Median additional cost per case for service contracts is $8040.40.

When total costs for all robotic system purchases, leases, and system upgrades are divided by overall case volume, an addition $1538.86 per case can be added to costs related to using the robotic system.

Total costs related to using the robotic system includes median costs for disposable supplies of $1519, nondisposable instruments = $2242.60, service contracts = $8040.40, and systems = $1538.86, for total median cost of $13,340.86 per case related to using the robotic system.

#### Admissions costs

The average cost per day for an admission following an open or laparoscopic surgery was equivalent. The average cost per day for admission following a robotic operation was 1.7 times greater than open or laparoscopic, largely in part due to higher operating room costs. These costs included supplies but did not include costs for robotic systems or service contracts. When you take into account the shorter LOS associated with robotic and laparoscopic cases, the cost of admission following a laparoscopic operation is the lowest. The cost of admission following a robotic operation is 1.5 times the cost of a laparoscopic operation, and the cost of admission following an open operation is 2.1 times that of a laparoscopic operation.

## Discussion

The utilization of robotic surgery at our institution has increased significantly over a period of just over 10 years. In 2006, there were 12 surgeons across 3 specialties performing robotic surgery, for a total of 120 cases. In contrast, in 2016 there were 28 surgeons across 5 specialties that performed a total of 586 cases. We did see a decrease in case volume in 2013 and 2014 related to a change in department leadership and the transition of some high-volume robotic surgeons to different institutions, but overall the program has grown tremendously year after year and we anticipate a continued increase in robotic case volume.

With increases in the number of cases, we have observed an overall decrease in case times as OR personnel have become more familiar with the technology. This was observed despite increased complexity of the patients undergoing robotic surgery, as demonstrated by an overall increase in patient ASA class during the study time period. However, when individual operations were analyzed they did not all show clear improvement in efficiency. Many operations had the longest average case times a couple years after the first case was performed. We believe this to be a reflection of performing more complicated cases in patients with more comorbidities, and lower likelihood of converting to open even in these more complex operations with acquisition of more advanced robotic skills.

In addition to surgeon learning curve with the technology, there are a few other factors that may have contributed to improved efficiency at our institution. At the beginning of the robotic program, additional monitoring, such as arterial lines, was used for patients undergoing robotic procedures. With increased comfort with the platform and proven safety, these measures have no longer been performed routinely and have saved time in the OR. As newer robotic systems have been developed, docking and instrument exchanges have become more efficient. Dedicated nursing teams for robotic cases have been used to ensure proper training and improve efficiency with docking/undocking and instrument exchanges in the OR. Our institution also developed a formal robotic surgery curriculum for residents, which uses simulation and animal labs to improve familiarity with the equipment prior to using the robot in the OR.

Even over the last 4 years of this study, when most surgeons had been using the platform for several years, we noticed an improvement in case times. While a clear improvement in efficiency may not be observed in every operation every year, overall efficiency of the robotic program as a whole has improved. Improvements in OR time decreases costs and allows more time for additional cases to be performed, increasing revenue.

We have observed a larger variety of cases, more complex cases in patients with more comorbidities, and multi-specialty cases with lower rates of conversion to open as surgeons have become proficient with the surgical platform. Few other surgical technological advancements have observed such rapid growth. Despite the wide variety in type of cases and specialties using the robot, the majority of cases have been performed by a small number of surgeons who predominantly perform operations within the pelvis.

Increased costs are a significant source of discussion and controversy when it comes to robotic-assisted surgery. It is very difficult to perform cost analysis when so many factors are involved. We attempted to include many of these factors by analyzing supply costs, length of stay, conversions to open, readmissions, and costs related to purchasing systems and service contracts. Some costs are difficult to offset, such as the increased costs associated with robotic surgical supplies. Barbash et al. [6] examined all cost studies of robot-assisted procedures published from 2005 to 2010 and found that, on average, the additional cost of using a robot for an operation was about $1600. This was very similar to the $1519 average cost of robotic disposable supplies per case that we found in our study. However, we calculated an additional $11,818 in robot-related costs that could be added to each case when costs for instruments, repairs, service contracts, and systems are included. As case volume increases, these costs can be spread out over a larger number of operations and average cost per case for these fees and purchases decreases.

There are many other factors associated with cost analysis that are hard to quantify. It is difficult to know whether the presence of a robot in a medical system is a factor that may recruit more patients, leading to increased operative volume and therefore revenue. Although they are becoming common throughout large medical centers, robotic systems are often viewed as a marker of cutting-edge patient care and technology, and might be purchased in order to remain competitive with other health systems. Hospitals were more likely to acquire a robot if they were in areas where a higher proportion of surrounding hospitals had a robot [7].

When looking at the costs or outcomes of all robotic operations compared to open or laparoscopic, the results are comparable or favorable. Similar to the published literature, we have shown improved short-term outcomes compared to laparoscopic surgery with regard to readmissions and length of stay, but long operative times and high costs [8–10].

Most studies on robotic surgery have looked at outcomes or costs for a specific type of operation. However, when investing in robotic surgery equipment and infrastructure, the investment is usually not for a specific operation or an individual surgeon, but it is an investment for the entire department. Therefore, this study may be helpful for institutions that are considering investing in a robotic surgery program, as it shows how the program as a whole has changed and grown over time, and gives insight into what they may experience. There is unlikely to be a linear decrease in operative time every year, however, as the program matures and surgeons and OR staff become more experienced, one can expect to see overall improvements in efficiency over time. A program may also expect to see increased volume, more complex cases, and benefits in regard to length of stay and overall costs related to hospitalizations.

A disadvantage of this study is that the data are retrospective and observational. The surgical platform was chosen by the attending surgeon on a case-by-case basis. Factors, such as medical comorbidities, disease severity, body habitus, surgical history, and even availability of the robotic system, all go into consideration when choosing what surgical platform to use. Therefore, the individual cases inherently have selection bias and cannot be equally compared to each other.

Finally, there are benefits of robotic surgery that cannot be quantified by data analysis. As shown in our study, the majority of robotic operations are performed in the pelvis. The robot provides exposure and access into areas that can be extremely difficult to operate in with open or laparoscopic techniques. The robotic system also provides improved ergonomics over traditional approaches, which in long-term follow-up may prove to have significant impacts on quality of life and careers for surgeons [11].

The robotic surgery program at our institution has observed increases in use across specialties, a wide variety of procedures performed, and outcomes that are equivalent or superior to those observed with traditional surgical platforms. While costs remain high, these are offset by improved outcomes with regard to conversion to open, length of stay, and admissions costs. While significant conclusions regarding robotic surgery programs as a whole cannot be made from this single institution retrospective study, it is interesting to see how utilization of this new technology has grown and changed over a short period of time. It will be interesting to see how surgical programs continue to change as new technology becomes available.
